# Time-Restricted Feeding Improved Vascular Endothelial Function in a High-Fat Diet-Induced Obesity Rat Model

**DOI:** 10.3390/vetsci9050217

**Published:** 2022-04-28

**Authors:** Ahmad Khusairi Azemi, Abdul Rahim Siti-Sarah, Siti Safiah Mokhtar, Aida Hanum Ghulam Rasool

**Affiliations:** 1 Department of Pharmacology, School of Medical Sciences, Health Campus, Universiti Sains Malaysia, Kota Bharu 16150, Kelantan, Malaysia; madkucai89@gmail.com (A.K.A.); safiahm@usm.my (S.S.M.); 2 Hospital Universiti Sains Malaysia, Kubang Kerian, Kota Bharu 16150, Kelantan, Malaysia; sarahwong901@gmail.com

**Keywords:** time-restricted feeding, eNOS, Akt, endothelial dysfunction, obesity, endothelium-dependent relaxation

## Abstract

Obesity, where there is enhancement of stored body fat in adipose tissues, is associated with cardiovascular complications that are mainly related to atherosclerosis. Time-restricted feeding (TRF) is a form of restricted eating aimed at reducing weight in obese subjects. The present study aims to investigate changes in vascular endothelial function, endothelial nitric oxide synthase (eNOS), and protein kinase B (Akt) protein expressions with TRF in obese and normal rats. Male Sprague Dawley rats were divided into two normal and three obese groups; obesity was induced in the obese groups by feeding with a high-fat diet (HFD) for six weeks. After six weeks, rats were equally divided into five groups (*n* = 7 per group): Normal group (NR) which continued on a standard diet for six more weeks, normal group switched to TRF with a standard diet for six weeks (NR + TRFSD), obese group (OR) which continued on HFD for six more weeks, obese group switched to TRF of HFD (OR + TRFHFD), and obese group switched to TRF of a standard diet (OR + TRFSD). TRF was practiced for six weeks, after which the rats were sacrificed. Aortic endothelium-dependent and endothelium-independent relaxations and contractions were assessed using the organ bath. Aortic eNOS and Akt protein expressions were determined using immunoblotting. Fasting blood glucose, body weight, body mass index (BMI), serum lipid profile, Lee’s index, serum insulin levels, and sensitivity (HOMA-IR) were also measured. Endothelium-dependent relaxation was significantly impaired, while endothelium-dependent contraction increased in obese rats compared to that in normal rats. Both obese groups which underwent TRF with a HFD and standard diet improved their impairments in endothelium-dependent relaxation and reduced endothelium-dependent contraction; these were associated with increased expressions of aortic eNOS and Akt protein. Both obese groups with TRF reduced body weight, BMI, Lee’s index, total cholesterol, triglycerides, low-density lipoprotein cholesterol, and improved insulin sensitivity. TRF improved endothelium-dependent relaxation and reduced endothelium-dependent contraction, thus attenuating endothelial dysfunction in obese rats. These were associated with increased aortic eNOS and Akt protein expressions.

## 1. Introduction

Cardiovascular disease is a leading cause of morbidity and mortality worldwide; accounting for almost one-third of all deaths [1,2]. Being overweight and obese are identified as major contributors to cardiovascular diseases and other chronic diseases with cardiovascular complications such as diabetes [3].

Obesity occurs due to upregulation of appetite and/or reduced calorie utilization by governing cellular function and physical activity. This dysregulation leads to the formation of excess adipocytes which increases cytokine release; one consequence of this is the occurrence of vascular complications [4]. These vascular complications are often associated with hyperlipidemia, endothelial dysfunction, and atherosclerosis. Hence, the management of obesity is beneficial to prevent and counteract these comorbidities [4,5]. 

Nowadays, diet modification represents a key factor to enhance the health status and welfare of animals; indeed, within the scientific community; diet alterations have been widely accepted as a useful strategy to modulate and/or optimize the biochemical and molecular pathways which orchestrate the metabolic responses of the animals to both physiological and pathological conditions [6,7].

Emerging evidence suggests that periodic fasting can improve various measures of health and longevity. Time-restricted feeding (TRF) involves limiting daily food intake to a maximum period of 10 h, followed by a daily fast of at least 14 h [8,9]. TRF has received keen interest from the public as an alternative to the traditional daily continuous energy restriction model for the management of obesity and its related disorders [2]. In rodents, TRF has been reported to reduce body weight and hyperlipidemia, improve glycemic control, increase energy expenditure, lower insulin levels, reduce hepatic fat, and reduce serum inflammatory markers [10,11,12,13,14,15,16,17,18]. In humans, TRF has been shown to reduce body weight, reduce fasting glucose and insulins levels, reduce insulin resistance, dyslipidemia, and inflammation marker in the blood [9,19,20].

Despite the beneficial effects of fasting in reducing weight, the impact of regular TRF on the vasculature, specifically endothelial function remains unclear. Endothelial function plays a pivotal role in the regulation of vascular tone and tissue blood flow, maintaining blood fluidity, and regulating inflammatory responses [21,22]. In this study, we hypothesized that TRF for six weeks in HFD-induced obese rats would improve vascular endothelial function parameters.

## 2. Materials and Methods

### 2.1. Preparation of High-Fat Diet

A high-fat diet (HFD) was prepared according to a method described previously [23]. The HFD was prepared with a mixture of 50% (*w*/*w*) standard diet (Gold Coin Feedmils, Port Klang, Malaysia), 38% (*w*/*w*) ghee, 8% (*w*/*w*) full-cream milk, and 4% (*w*/*w*) white sugar. Table 1 showed the composition of HFD used in the present study.

### 2.2. Animals and Experimental Protocols

The experimental protocol used in this study was approved by the Universiti Sains Malaysia (USM) Institutional Animal Care and Use Committee (USM IACUC) with voucher number [USM/IACUC/2020/(126)(1111)]. Thirty-five male Sprague Dawley rats (12 weeks old; 250–300 g) were used in the study and were then divided into normal and obese groups. Throughout the experiment, rats were housed in groups of 2–3 rats in polypropylene cages in a well-ventilated animal room at a temperature of 25 ± 2 °C with a 12 h light/dark cycle. The normal group was fed a standard diet, while the obese group was fed a HFD. After six weeks, the rats were randomly selected and assigned to 5 groups of 7 rats each. The grouping of rats was:

Group 1: Rats fed a standard diet for 6 weeks, then continued for 6 weeks on a standard diet (NR)
Group 2: Rats fed a standard diet for 6 weeks, then 6 weeks of TRF with a standard diet (NR + TRFSD)
Group 3: Rats fed a HFD for 6 weeks, then continued for 6 weeks on HFD (OR)
Group 4: Rats fed a HFD for 6 weeks, then 6 weeks of TRF with a HFD (OR + TRFHFD)
Group 5: Rats fed a HFD for 6 weeks, then 6 weeks TRF with a standard diet (OR + TRFSD)

Rats that underwent TRF were provided their respective diet for 8 h only to be consumed in a day (9.00 a.m.–5.00 p.m.), and thereafter, food was withheld for 16 h (5.00 p.m.–9.00 a.m. the next day). All rats, however, had free access to water. For non-TRF groups (NR and OR groups), food was provided *ad libitum*, available to be eaten for 24 h. Diets were given in the same standardized amount daily for all groups, however, the groups undergoing TRF could only consume them within an 8 h duration per day. At the end of the twelfth week, the rats were euthanized by intraperitoneal injection of a combination of ketamine (300 mg/kg) and xylazine (30 mg/kg) [23]. A blood sample from the renal artery was collected from each rat and placed in standard vacutainer tubes. Blood samples (serum) were used for biochemical measurements.

### 2.3. Biochemical Measurements of Serum Lipid Profile and Atherogenic Index

The blood sample was centrifuged at 1500 g (Kubota 4000, Tokyo, Japan) for 25 min, and the resulting serum used for lipid profile analyses. Serum total cholesterol (TC), triglycerides (TG), and high-density lipoprotein-cholesterol (HDL-C) were measured using the colorimetric method with Integra 800 automatic immunoanalyzer (Roche Diagnostic Sytems, Manheim, Germany). The serum LDL-C was calculated using Friedwald formula [24], as follows:

LDL-C = TC − (HDL-C + TG/5)

The atherogenic index (AI) was calculated based on the LDL-C and HDL-C values in the serum [24] as follows:

AI = LDL-C/HDL-C

### 2.4. Anthropometric Measurements

In the present study, the body mass index (BMI) and Lee’s index were determined. BMI is defined as body weight divided by the square of the body length (nose to anal length in rodents) [25,26]. Lee’s index can be used as a reliable way to determine obesity in rats [26,27].

The BMI and Lee’s index were calculated as follows:

(i) BMI = body weight (g)/length^2^ (cm^2^)
(ii) Lee’s index = cube root of body weight (g)/nose to anus length (cm)

### 2.5. Serum Insulin Level and Homeostatic Model Assessment for Insulin Resistance (HOMA-IR) 

Blood samples were centrifuged at 1500× *g* (Kubota 4000, Tokyo, Japan) for 25 min, and the resulting serum was used for insulin level measurement. Serum insulin level was measured using an ELISA kit according to the manufacturers’ instructions (Elabscience, Houston, TX, USA; Catalog No. E-EL-R3034). Based on the ELISA kit’s instructions, the dilution factor used for serum samples was 1:10. The serum samples were diluted in a sample diluent provided with the ELISA kit. Insulin resistance was assessed by HOMA-IR, a mathematical model describing the degree of insulin resistance from fasting blood glucose and insulin as described previously [28,29].

### 2.6. Macrovascular Function Studies

Macrovascular endothelial function was conducted using an isolated organ bath. After the rats were sacrificed, the thoracic aorta was immediately isolated and placed in a petri dish containing ice-cold and oxygenated (95% O_2_ and 5% CO_2_) physiological saline solution (composition, mM): sodium chloride (NaCl, 118); sodium hydrogen carbonate (NaHCO_3_, 250; potassium chloride (KCl, 4.7); potassium dihydrogen phosphate (KH_2_PO_4_, 1.2); magnesium sulfate heptahydrate (MgSO_4_·H_2_O, 1.18); calcium chloride dihydrate (CaCl_2_.2H_2_O, 2.0); D-glucose, (5.5). The thoracic aorta (approximately 16–20 mm in length) was dissected and cleared of adhering tissues and fat. Arteries were then cut into ring segments of 3 mm in length. In some preparations, the endothelial cells were removed by scrubbing the intimal surface of the artery segments with blunt forceps. Each aortic segment was vertically fixed between hooks in an organ bath chamber (10 mL capacity) containing physiological saline solution. The bathing solution was maintained at 37 °C (pH 7.4) and aerated with carbogen gas (95% O_2_ and 5% CO_2_). The upper end of the ring segment was connected to a force-displacement transducer (ADInstruments, Bella Vista, Australia). The ring segment was first stretched to an optimal resting tension of 1.0 g and was equilibrated for 1 h before commencement of the experimental protocol. The ring segment was first exposed to potassium chloride (KCl; 60 mM) to obtain a reference contraction and to ensure smooth muscle viability. Then, the ring segment was precontracted with phenylephrine (10^−6^ M) followed by relaxation to acetylcholine (ACh, 10^−6^ M), to test for the integrity of the endothelial cells. To study endothelium-dependent relaxation, aortic ring segments with endothelium were exposed to phenylephrine (10^−6^ M) and exposed to cumulative concentrations of ACh (10^−9^ to 10^−4^ M). For endothelium-independent relaxation, the aortic ring segments were exposed to phenylephrine (10^−6^ M) followed by cumulative addition of sodium nitroprusside (10^−9^ to 10^−4^ M). For endothelium-dependent contraction, ring segments with endothelium were exposed to a cumulative concentration of calcium ionophore (10^−9^ to 10^−4^ M) in the presence of L-Nitro-Arginine Methyl Ester (L-NAME; 10^−4^ M) to inhibit endothelial NO synthase. Cumulative concentrations of phenylephrine (10^−9^ to 10^−4^ M) were used to assess endothelium-independent contraction [30,31]. Changes in tension induced by calcium ionophore and phenylephrine were expressed as percentage of the reference contraction to 60 mM KCl, obtained at the beginning of the experiment.

### 2.7. Immunoblotting

Immunoblotting was conducted according to the protocol previously described by Azemi et al. (2020) [30]. The remaining left thoracic aorta (approximately 10–14 mm in length) which had not been used for the functional study were used for immunoblotting. Aortas from all seven rats in each group were used for immunoblotting. The thoracic aorta was first lysed using a RIPA lysis buffer with a protease inhibitor cocktail of 0.05% (Sigma Chemical Co., St. Louis, MO, USA). The total protein concentration was then measured using a protein determination kit (Cayman Chemicals, Ann Arbor, MI, USA). About 30 μg of total protein was then loaded and separated on 10% sodium dodecyl sulfate (SDS)-polyacrylamide gel and then transferred to polyvinylidene difluoride (PVDF) membranes (Millipore Corp., Billerica, MA, USA). The membranes were then blocked with 5% non-fat skimmed milk at room temperature for 60 min and then incubated with the primary antibodies for eNOS (1:1000), Akt (1:1000), and β-actin (1:1000) (Cell Signaling, Danvers, MA, USA). After washing with tris buffer saline, the membranes were incubated with horseradish peroxidase (HRP)-conjugated polyclonal secondary antibody (1:1000; Cell Signaling, Danvers, MA, USA) in a blocking buffer. All proteins were detected using Chemi-Lumi One Series (Nacalai Tesque, Kyoto, Japan) with FlourChem M (ProteinSimple, San Jose, CA, USA) imaging systems and quantified using ImageJ software (https://imagej.nih.gov/ij/download.html, accessed on 16 August 2021). The relative presence of eNOS and Akt were expressed as the percentage of the total amount of protein (indicated by the intensity of protein band for β-actin) in the same animal sample [31]. 

### 2.8. Statistical Analysis

All data were expressed as mean ± standard error of the mean (SEM). Statistical analysis of the data was performed using GraphPad Prism software version 8.0 (GraphPad Software, San Diego, CA, USA). Differences between the groups were analyzed using one-way analysis of variance (ANOVA) followed by Bonferroni’s post hoc test. Statistical significance was defined when the *p*-value was less than 0.05 (*p* < 0.05). 

## 3. Results

### 3.1. Effect of TRF on Body Weight, Fasting Blood Glucose, Serum Lipid Profile, and Atherogenic Index in the Obese Rat Model

Body weight and fasting blood glucose (FBG) levels of rats in the five study groups were shown in Table 2. No significant difference was seen in the initial body weight among the study groups. However, six weeks after administration of HFD, all groups fed the HFD demonstrated significant weight gains compared to their initial body weight. The OR group showed significant weight gain compared to that of the NR group after six weeks of HFD. The final body weight of rats in the OR group was significantly higher compared to that of the NR group (*p* < 0.001). Time-restricted feeding with a HFD (OR + TRFHFD) and a standard diet (OR + TRFSD) for six weeks in both obese groups significantly reduced body weight at the end of the study period compared to that of the OR group (Table 2). There was no significant difference in final FBG among the study groups at week 12.

In the present study, the levels of TC, TG, LDL-C, and AI were significantly higher in the OR group compared with that in the NR group (*p* < 0.05) (Table 2). The HDL-C level was significantly lower in the OR group compared to that in the NR group. Six weeks of TRF administration with a HFD in obese rats significantly reduced TG, LDL-C, and AI levels compared to those in the obese control group (OR) (Table 1). TRF with a standard diet for six weeks in obese rats significantly reduced TC, TG, LDL-C, and AI levels compared to those in the obese control rats (OR). TRF with a standard diet in normal rats significantly reduced TG, LDL-C, AI, and increased HDL-C levels compared to those in the obese control group (OR).

### 3.2. Anthropometric Measurements

The BMI of rats in the OR group was significantly higher compared to that of rats in the NR group (*p* < 0.0001). Six weeks of TRF significantly reduced the BMI in both OR + TRFHFD and OR + TRFSD groups compared that in the OR group (Table 3). 

Lee’s index of rats in the OR group was significantly higher compared to that of rats in the NR group. Time-restricted feeding for six weeks in the obese group fed a HFD (OR + TRFHFD) and the obese group fed a standard diet (OR + TRFSD) lowered Lee’s index compared to that of the obese control group (OR) (Table 3).

### 3.3. Effect of TRF on Insulin Levels and HOMA-IR

**Figure 1:**
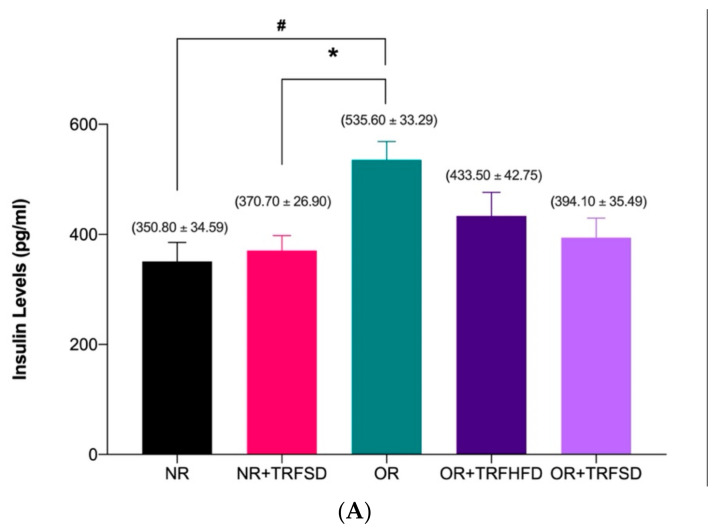

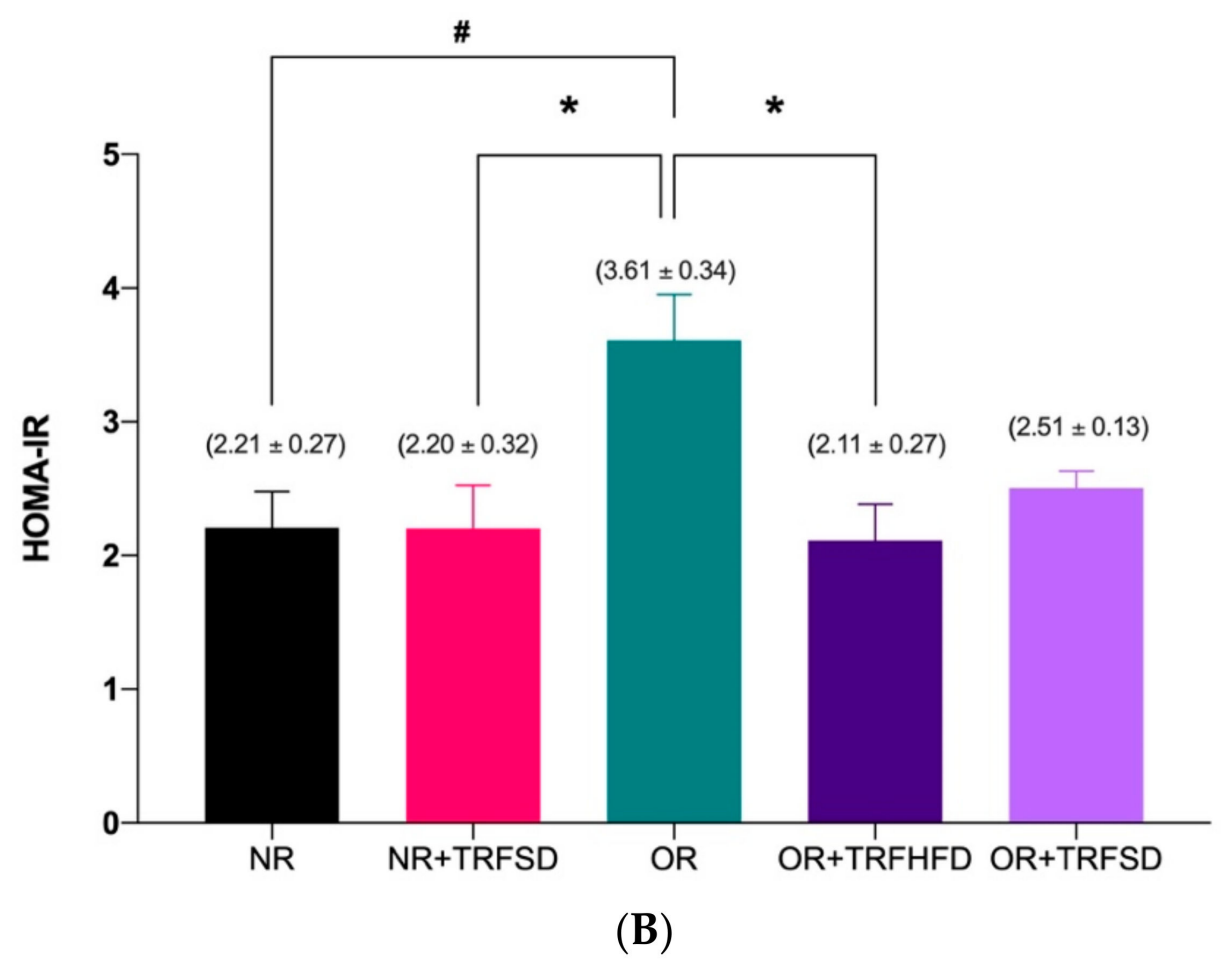
Effect of time-restricted feeding on serum insulin level (**A**) and HOMA-IR (**B**) in the normal and obese groups. Data are presented as mean ± SEM (*n* = 7). ^#^ *p* < 0.05 vs. NR. * *p* < 0.05 vs. OR.

Serum insulin levels were significantly higher in the OR group compared to that in the NR group (OR: 535.60 ± 33.29 vs. NR: 350.80 ± 34.59 pg/mL; *p* = 0.0460) (Figure 1A). Time-restricted feeding for six weeks in the obese group fed a HFD (OR + TRFHFD) and the obese group fed a standard diet (OR + TRFSD) did not affect the serum insulin levels compared to the obese control group (OR) (Figure 1A). However, six weeks with TRF in normal rats fed a standard diet (NR + TRFSD) significantly reduced insulin levels compared to those in the obese control group (NR + TRFSD: 370.70 ± 26.90 vs. OR: 535.60 ± 33.29 pg/mL; *p* = 0.0327).

Insulin resistance (HOMA-IR) in the OR group was significantly higher (OR: 3.61 ± 0.34 vs. NR: 2.21 ± 0.27; *p* = 0.0253) compared to that in the NR group (Figure 1B). Six weeks of TRF in the obese group fed a HFD (OR + TRFHFD) significantly reduced (OR + TRFHFD: 2.11 ± 0.27 vs. OR: 3.61 ± 0.34; *p* = 0.0159) insulin resistance compared to the that of the obese control group (OR). However, no significant difference was seen between OR + TRFSD and OR groups (*p* = 0.0995) in their insulin resistance. Other than that, six weeks of TRF with a standard diet in the NR group (NR + TRFSD) significantly lowered insulin resistance (HOMA-IR) compared to that of the OR group (NR + TRFSD: 2.20 ± 0.32 vs. OR: 3.61 ± 0.34; *p* = 0.0171).

### 3.4. Vascular Function Study

#### 3.4.1. Contractile Response to KCl

**Figure 2:**
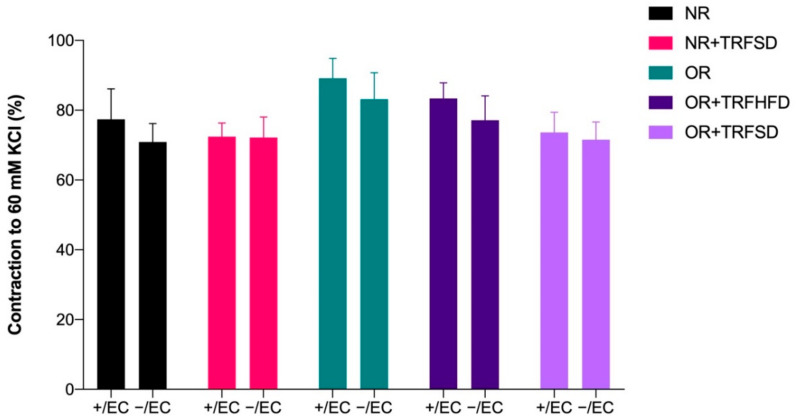
Contractions to KCl (60 mM) in thoracic aorta of rats. + /EC: endothelium-dependent; −/EC: endothelium-independent.

There were no significant differences in contractions to KCl (60 mM) between study groups (Figure 2). 

#### 3.4.2. Effect of TRF on Vascular Relaxations in Normal and Obese Rats

**Figure 3:**
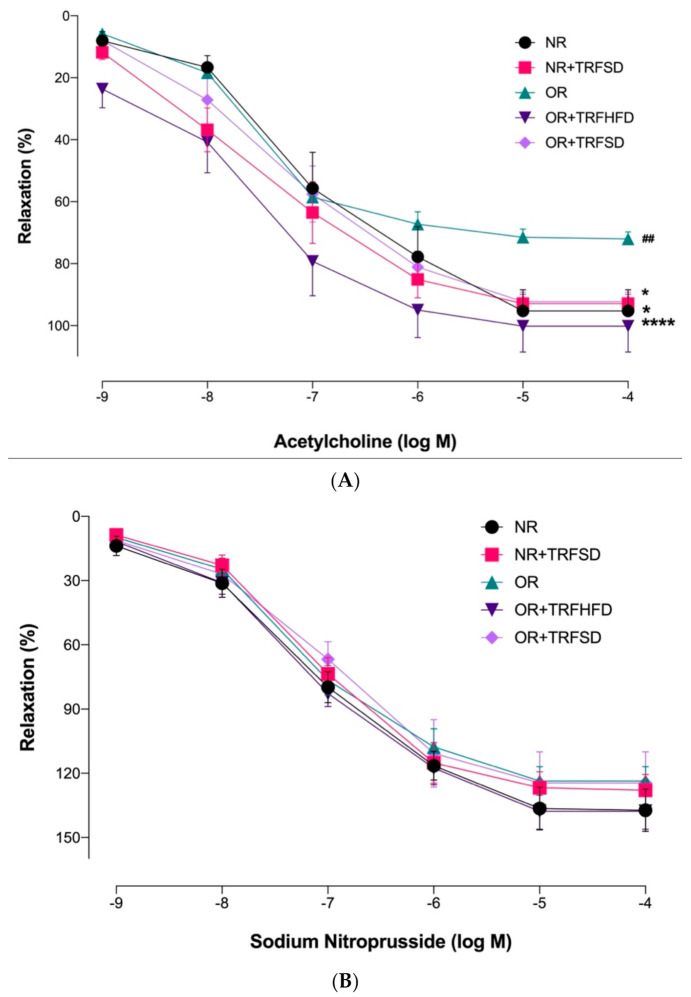
Endothelium-dependent and endothelium-independent relaxations in the thoracic aorta of rats: (**A**) Concentration-response curves to ACh (10^−9^–10^−4^ M); (**B**) Concentration-response curves to sodium nitroprusside (10^−9^–10^−4^ M). Data are presented as mean ± SEM (*n* = 7). Relaxations were expressed as a percentage of the contraction induced by phenylephrine (10^−6^ M). ^##^ *p* < 0.01 vs. NR. * *p* < 0.05 and **** *p* < 0.0001 vs. OR.

Endothelium-dependent relaxations to ACh were significantly reduced in the OR group compared to those in the NR group (*p* = 0.0044) (Figure 2, Table 3). No significant difference was seen between the NR + TRFSD group and the NR group. Higher maximum response (*E_max_*) to ACh in both OR + TRFHFD (*p* < 0.0001) and OR + TRFSD (*p* = 0.0178) groups represented improved endothelium-dependent relaxation compared to that of the OR group (Figure 3, Table 4). In addition, TRF in the NR + TRFSD group also showed higher endothelium-dependent relaxation compared to that in the OR group (*p* = 0.0108).

Endothelium-independent relaxations to sodium nitroprusside were comparable among the study groups; (Figure 3, Table 4).

#### 3.4.3. Effect of TRF on Vascular Contractions in Normal and Obese Rats 

**Figure 4:**
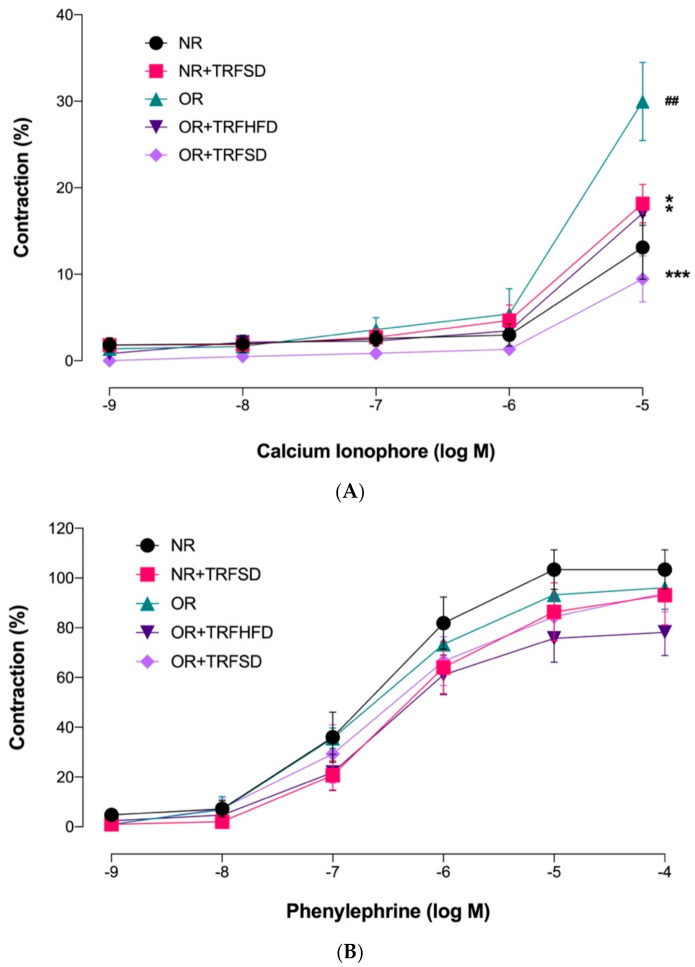
Endothelium-dependent and endothelium-independent contractions in the thoracic aorta of rats. (**A**) Concentration-response curves to calcium ionophore (10^−9^−10^−5^ M); (**B**) Concentration-response curves to phenylephrine (10^−9^–10^−4^ M). Data are presented as mean ± SEM (*n* = 7). ^##^ *p* < 0.01 vs. NR. ** *p* < 0.05 and *** *p* < 0.001 vs. OR.

OR group showed a higher *E_max_* value in response to calcium ionophore compared to that of the NR group, indicating that obesity increased endothelium-dependent contraction (*p* = 0.0062) (Figure 4, Table 5). OR + TRFHFD (*p* = 0.0186) and OR + TRFSD (*p* = 0.0003) groups showed lower endothelium-dependent contractions to calcium ionophore compared to those in the OR group. In addition, TRF in the NR + TRFSD group showed lower endothelium-dependent contraction compared to that in the OR group (*p* = 0.0133). Time-restricted feeding with the standard diet for six weeks in normal rats showed no significant difference in endothelium-dependent contraction compared to normal rats (NR). 

Endothelium-independent contractions to phenylephrine were comparable in all study groups; (Figure 4, Table 5).

### 3.5. Immunoblotting

**Figure 5:**
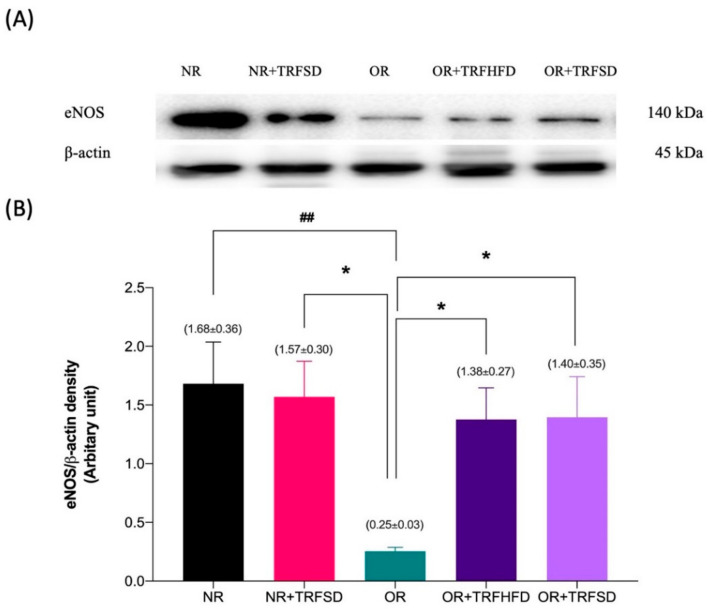
Immunoblot analysis of eNOS protein expression in the thoracic aorta: (**A**) Representative immunoblot blot showing the expression of eNOS protein or NR, NR + TRFSD, OR, OR + TRFHFD, and OR + TRFSD groups; (**B**) Graphical representation of the data, normalized to β-actin. Data are presented as mean ± SEM (*n* = 7). ^##^ *p* < 0.01 vs. NR. * *p* < 0.05 vs. OR.

The expression of eNOS protein was 6.72-fold lower in the thoracic aorta of rats in the OR group compared to that in the NR group (OR: 0.25 ± 0.03 vs. NR: 1.68 ± 0.36 arbitrary units (au); *p* = 0.0073). The expressions of eNOS protein were 5.52-fold and 5.60-fold higher in the thoracic aorta of rats in both OR + TRFHFD (OR + TRFHFD: 1.38 ± 0.27 vs. OR: 0.25 ± 0.03 au; *p* = 0.0365) and OR + TRFSD (OR + TRFSD: 1.40 ± 0.35 vs. OR: 0.25 ± 0.03 au; *p* = 0.0325) groups compared to that in the OR group (Figure 5B). Figures in supplementary Files showed immunoblot of eNOS, Akt and β-actin protein expression. The expressions of eNOS protein in the NR + TRFSD group were 6.28-fold higher compared to that in the OR group (NR + TRFSD: 1.57 ± 0.30 vs. OR: 0.25 ± 0.03 au; *p* = 0.0150). No difference was seen between NR + TRFSD and NR groups in their eNOS levels.

**Figure 6:**
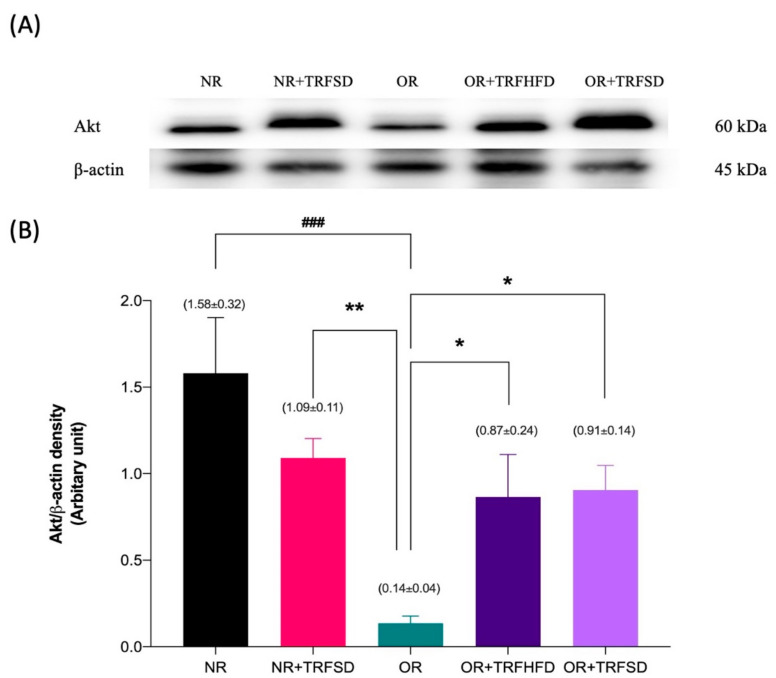
Immunoblot analysis of Akt protein expression in the thoracic aorta: (**A**) Representative immunoblot showing the expression of Akt protein or NR, NR + TRFSD, OR, OR + TRFHFD, and OR + TRFSD groups; (**B**) Graphical representation of the data, normalized to β-actin. Data are presented as mean ± SEM (*n* = 7). ^###^ *p* < 0.001 vs. NR. * *p* < 0.05, ** *p* < 0.01 vs. OR.

The expression of Akt protein was 11.29-fold lower in the thoracic aorta of rats in the OR group compared to that in the NR group (OR: 0.14 ± 0.04 vs. NR: 1.58 ± 0.32 au; *p* = 0.0003). The expressions of Akt protein were 6.21-fold and 6.50-fold higher in the thoracic aorta of rats in both OR + TRFHFD (OR + TRFHFD: 0.87 ± 0.24 vs. OR: 0.14 ± 0.04 au; *p* = 0.0488) and OR + TRFSD (OR + TRFSD: 0.91 ± 0.14 vs. OR: 0.14 ± 0.04 au; *p* = 0.0471) groups compared to that in the OR group (Figure 6B). The expressions of Akt in the NR + TRFSD group were 7.79-fold higher compared to that in the OR group (NR + TRFSD: 1.09 ± 0.11 vs. OR: 0.14 ± 0.04 au; *p* = 0.0097). No difference was seen between NR + TRFSD and NR groups in their Akt levels.

## 4. Discussion

Limited studies had investigated the impact of time-restricted feeding (TRF) on cardiovascular parameters in animal models. A few studies indicate that TRF reduces the risk factors for cardiovascular diseases, such as serum lipid profile and inflammatory cytokines in rats [2,32]. To the best of our knowledge, this current study is the first to show that TRF improved endothelium-dependent relaxation and contraction, which were associated with increased eNOS and Akt protein expressions in the aorta of HFD-induced obese rats. This study also demonstrated that TRF reduced body weight, BMI, Lee’s index, TC, TG, LDL-C, atherogenic index (AI), and increased insulin sensitivity in obese rats.

In the present study, feeding a HFD led to obesity (weight gain) in rats as compared to rats fed with a standard commercial food pellet (normal rats). This result was similar to the data recorded by previous studies [33,34,35]. In the present study, six weeks of TRF with a HFD and a standard diet in the obese rats significantly reduced the body weight of rats compared to that of control obese rats. Similar findings have been reported previously in different animal strains with TRF [17,36,37]. In addition, these findings in animal models are similar to those reported by studies in obese humans who underwent TRF [9,38], although a few studies failed to show this effect [39,40]. In the present study, TRF with both a HFD and a standard diet for six weeks significantly reduced BMI and Lee’s index in obese rats compared to those in control obese rats. However, TRF with a standard diet in the normal rats did not affect body weight, BMI, and Lee’s index compared to ad libitum-fed normal rats (NR). 

It has been reported that HFD-induced hyperlipidemia results in a significant increase in serum TC, TG, LDL-C, and AI [23,24,41]. The present study demonstrated that TRF for six weeks reduced the levels of TC, TG, LDL-C, and AI in obese rats. However, TRF did not affect HDL-C levels in obese rats. These findings are similar to data reported in the previous studies [32,42,43].

In the present study, twelve weeks of HFD feeding in obese rats significantly increased insulin levels; a finding similar to that reported in previous studies [28,44]. However, six weeks of TRF in both obese groups (OR + TRFHFD and OR + TRFSD) did not affect insulin levels. No animal studies are available for comparison for this parameter, however, this effect has been reported in human subjects practicing TRF [45,46,47]. The current study also showed that insulin resistance (HOMA-IR) was significantly higher in OR groups compared to that in normal groups; similar findings were reported in previous studies [28,29]. TRF for six weeks in the obese group fed a HFD significantly reduced insulin resistance compared to that in the obese control group. This effect was similar to that in studies reported previously in rats [43] and human subjects [9,48]. 

In this study, relaxation to ACh reflected endothelium-dependent relaxation. ACh binds to the muscarinic receptors on endothelial cells, resulting in the generation of diacylglycerol and inositol triphosphate (IP_3_). IP_3_ activates its receptor on the endoplasmic reticulum membrane, which induces Ca^2+^ release from the endoplasmic reticulum store. The resultant increase in intracellular Ca^2+^ increases eNOS activity, promoting NO production. Increased NO production diffuses to the underlying vascular smooth muscle and activates guanylyl cyclase to generate cyclic guanosine monophosphate (cGMP), promoting smooth muscle relaxation. Endothelial production of NO requires the formation of eNOS homodimers and phosphorylation of specific eNOS residues by protein kinase b (Akt) [49,50]. Akt directly phosphorylates eNOS at the site of eNOS phosphorylation, serine-1197 (S1197), enhancing its enzymatic activity and altering the sensitivity of the enzyme to Ca^2+^ [49,51]. Akt interacts with Hsp90, a protein that associates with and activates eNOS. Hsp90 serves as a scaffolding function to facilitate Akt-mediated phosphorylation of eNOS in the caveolae [51,52,53]. Since NO is an important regulator of vasomotor tone, the effects of Akt on eNOS phosphorylation and activation can significantly enhance vasorelaxation. Inhibition of Akt attenuates endothelium-dependent vasodilatation in response to acetylcholine [51,54]. 

In the present study, HFD-induced obese rats demonstrated lower aortic endothelium-dependent relaxation; a finding which agrees with the results from previous studies [41,55,56]. Our results showed that reduced ACh-induced endothelium-dependent relaxation was associated with lower eNOS and Akt protein expressions in the aorta of obese control rats (OR). A few studies have also reported that obesity is associated with reduced vascular eNOS [57] and Akt [58,59] protein expressions in HFD-induced obese animals. The impairment of endothelium-dependent relaxation, eNOS, and Akt expressions may be due to increased oxidative stress and inflammation observed in obese rats [57,60]. It has been shown that the administration of a HFD to rats increases serum and aortic tissue oxidative stress and inflammation [57,61,62]. Administration of a HFD also induces hyperlipidemia, which increases free radical production and oxidative stress; these may be due to the release of free radicals from the mitochondrial electron transport chain [63]. When free radicals are increased, especially the superoxide anions, they react with NO to form peroxynitrite, thus impairing NO-mediated relaxation. Increases in LDL-C levels induced by HFD also lead to an increase in its oxidized form (oxLDL-C) due to the increase in free radicals with obesity [57]. OxLDL-C increases the synthesis of caveolin-1, which binds to eNOS protein, thus reducing eNOS activity and NO bioavailability [64,65]. In the current study, TRF for six weeks significantly increased endothelium-dependent relaxation in the aorta of obese rats. This was supported by higher expressions of eNOS and Akt proteins in the aorta of both groups (OR + TRFHFD and OR + TRFSD). In addition, no differences were seen in endothelium-dependent relaxation between both obese groups which underwent TRF and normal rats, indicating that TRF restored endothelial function of obese rats to normal values. This may be due to weight reduction, as the weights of both obese rats that underwent TRF became similar to that of normal rats at the end of six weeks of TRF.

A few mechanisms might contribute to the improvement of endothelium-dependent relaxation in HFD-induced obese rats with TRF. First, it could be due to the reduction of total cholesterol, triglycerides, and LDL-C. Reduced LDL-C levels may prevent caveolin-1 from binding to eNOS protein, thus improving NO generation and bioavailability. Secondly, reduced insulin resistance (HOMA-IR) with TRF might increase endothelium-dependent relaxation. Reduced insulin resistance leads to improved insulin sensitivity, which increases the activity of eNOS and Akt [66]. These were supported by higher aortic tissue expressions of eNOS and Akt in obese groups practicing TRF compared to those in the obese group not practicing TRF. These led to increase NO production and bioavailability, which improved vasodilation.

The response of vascular smooth muscle cell relaxation to sodium nitroprusside was tested without endothelium. There were no significant differences between the groups in their responses to sodium nitroprusside, indicating that endothelium-independent relaxation was not affected by TRF.

Calcium ionophore is used to increase intracellular Ca^2+^ levels in intact cells. It increases the transport of Ca^2+^ ions across the endothelial cell membrane into endothelial cells [67]. In studies assessing endothelial-dependent contraction, calcium ionophore-induced endothelial contractions were studied while simultaneously using L-NAME in the incubation of blood vessels. L-NAME inhibits the activity of eNOS, inhibiting endothelial NO production, which potentiates vascular contraction due to calcium ionophore. In the present study, twelve weeks of HFD-feeding in obese rats increased aortic endothelium-dependent contraction compared to that in normal rats. Both obese groups that underwent TRF for six weeks significantly reduced endothelium-dependent contraction. A few factors may contribute to the reduction of endothelium-dependent contraction in obese rats undergoing TRF. First, the reduction of contractile response might be due to the high eNOS and Akt protein expressions and augmented endothelium-dependent relaxation in obese rats with TRF. Increased eNOS and Akt protein expression improved NO production and bioavailability in endothelial cells, which reduced the production of endothelium-derived contracting factors (EDCF). Vanhoutte and Tang (2008) have demonstrated that NO inhibits the production and action of EDCF [68]. The inhibition of EDCF leads to reduced vascular smooth muscle contraction [68]. Secondly, reduction in endothelium-dependent contraction may be due to the reduction of insulin resistance. Reduced insulin resistance leads to increased insulin sensitivity, which stimulates the production of endothelial NO [69], thus preventing EDCF production [70]. 

Phenylephrine acts as a standard vasoconstrictor commonly used to induce endothelium-independent contraction. In the present study, there were no significant differences among the study groups in their responses to phenylephrine, indicating that smooth muscle contraction was not affected by TRF.

The effects of TRF on vascular structure and function, for example, on blood and vascular tissue oxidative stress and inflammation, could be further studied. Its effect on vascular structural changes and atherosclerosis will also be beneficial. In addition, the effects of TRF on other high cardiovascular risk animal models, such as diabetic models, should be further investigated.

## 5. Conclusions

This study showed that six weeks of TRF improved endothelium-dependent relaxation and reduced endothelium-dependent contraction in the aorta of high-fat-diet-induced obese rats. These were associated with enhanced eNOS and Akt protein expressions, reduced TC, TG, LDL-C, insulin resistance, and BMI.

## Figures and Tables

**Table 1:** Composition of HFD.

Parameter, Unit	g/100 g
Protein	6.10
Total fat	39.00
Total carbohydrate	46.50
Ash	3.90
Moisture	4.50
Energy, kcal/100 g	561 (2356 kJ)

**Table 2:** Body weight and FBG levels in normal and obese rats.

Title	NR	NR + TRFSD	OR	OR + TRFHFD	OR + TRFSD
Initial body weight (g) (Day 0)	315.30 ± 4.34	305.80 ± 6.25	326.0 ± 3.70	311.20 ± 11.49	309.80 ± 12.52
Body weight before TRF (g) (Week 6)	386.80 ± 8.43 ^a^	411.60 ± 2.99 ^a^	453.00 ± 12.36 ^##, a^	428.80 ± 11.06 ^a^	414.30 ± 16.43 ^a^
Final body weight after TRF (g) (Week 12)	417.70 ± 5.26 ^a,b^	406.60 ± 6.92 ****^,a^	524.80 ± 20.52 ^###,a,b^	395.00 ± 17.01 ****^,a^	380.80 ± 4.49 ****^,a^
Final FBG (mmol/L)	4.40 ± 0.14	4.50 ± 0.29	4.63 ± 0.16	4.61 ± 0.27	4.67 ± 0.19
Total cholesterol (TC)	3.49 ± 0.24	4.13 ± 0.18	4.53 ± 0.07 ^#^	4.10 ± 0.22	3.38 ± 0.34 *
Triglycerides (TG)	0.60 ± 0.01	0.64 ± 0.07 **	1.37 ± 0.24 ^##^	0.75 ± 0.10 *	0.56 ± 0.08 **
High-density lipoprotein cholesterol (HDL-C)	0.70 ± 0.05	0.68 ± 0.06 *	0.47 ± 0.04 ^#^	0.62 ± 0.04	0.55 ± 0.02
Low-density lipoprotein cholesterol (LDL-C)	0.06 ± 0.02	0.09 ± 0.05 **	0.50 ± 0.12 ^##^	0.17 ± 0.09 *	0.10 ± 0.06 **
Atherogenic index (AI)	0.18 ± 0.08	0.14 ± 0.07 **	0.70 ± 0.16 ^##^	0.01 ± 0.01 ***	0.19 ± 0.11 **

Data are expressed as mean ± SEM. *n* = 7. ^a^ *p* < 0.05 vs. initial body weight; ^b^ *p* < 0.05 vs. middle body weight (Week 6); ^##^ *p* < 0.01, ^###^ *p* < 0.001 vs. NR group; * *p* < 0.05, ** *p* < 0.01, *** *p* < 0.001, **** *p* < 0.0001 vs. OR group.

**Table 3:** Effect of intermittent fasting on BMI and Lee’s index in normal and obese rats.

Title	NR	NR + TRFSD	OR	OR + TRFHFD	OR + TRFSD
Body length (cm)	25.23 ± 0.15	25.36 ± 0.21	25.14 ± 0.09	24.64 ± 0.30	24.57 ± 0.35
BMI	0.66 ± 0.01	0.64 ± 0.01 ****	0.80 ± 0.03 ^####^	0.68 ± 0.03 ***	0.66 ± 0.01 ****
Lee’s index	0.81 ± 0.01	0.80 ± 0.01 ****	0.90 ± 0.01 ^####^	0.82 ± 0.02 ***	0.81 ± 0.01 ***

Data are expressed as mean ± SEM. *n* = 7. ^####^ *p* < 0.0001 vs. NR group. *** *p* < 0.001, **** *p* < 0.0001 vs OR group.

**Table 4:** Relaxation to ACh and sodium nitroprusside in the thoracic aorta.

Title	NR	NR + TRFSD	OR	OR + TRFHFD	OR + TRFSD
Acetylcholine *E_max_* (%)	95.27 ± 6.78	92.89 ± 2.92 *	69.24 ± 1.83 ^##^	107.50 ± 4.72 ****	92.27 ± 2.96 *
Sodium nitroprusside *E_max_* (%)	137.30 ± 9.76	127.90 ± 7.27	111.30 ± 4.94	137.80 ± 8.33	130.50 ± 15.84

Data are expressed as mean ± SEM, *n* = 7. ^##^ *p* < 0.01 vs. NR group; * *p* < 0.05, **** *p* < 0.0001 vs. OR group.

**Table 5:** Contractions to calcium ionophore and phenylephrine in the thoracic aorta.

Title	NR	NR + TRFSD	OR	OR + TRFHFD	OR + TRFSD
Calcium ionophore *E_max_* (%)	13.10 ± 3.68	15.40 ± 1.57 *	29.96 ± 4.51 ^##^	17.04 ± 1.39 *	9.45 ± 2.67 ***
Phenylephrine *E_max_* (%)	91.64 ± 3.48	86.45 ± 11.80	109.80 ± 8.60	78.16 ± 9.29	85.10 ± 7.15

Data are expressed as mean ± SEM. *n* = 7. ^##^ *p* < 0.01 vs. NR group; * *p* < 0.05, *** *p* < 0.001 vs. OR group.

## Data Availability

Data is available upon request.

## Supplementary Materials

The following are available online at https://www.mdpi.com/article/10.3390/vetsci9050217/s1, Immunoblot figures (uncropped blots) with densitometry reading/intensity ratio of each band in the present study.
