# Supplementary material for: Time-Restricted Feeding Improved Vascular Endothelial Function in a High-Fat Diet-Induced Obesity Rat Model
Source: Vet Sci. 2022 Apr 28;9(5):217. doi: 10.3390/vetsci9050217 (PMC9147025; doi:10.3390/vetsci9050217)
Supplement: Supplementary file 1 [file vetsci-09-00217-s001.zip › vetsci-1632223-supplementary.pdf]

Endothelial nitric oxide synthase (eNOS)

NR	NR+TRFSD	OR	OR+TRFHFD	OR+TRFSD	OR	NR	NR+TRFSD	OR

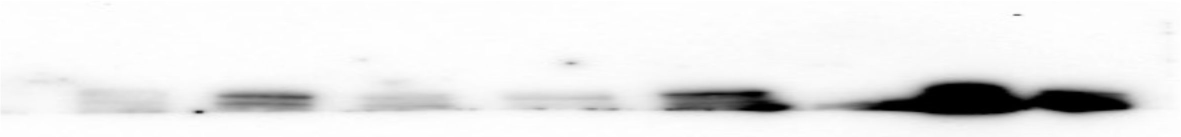

OR	OR+TRFHFD	OR+TRFSD	OR	NR	NR+TRFSD	OR	OR+TRFHFD	OR+TRFSD

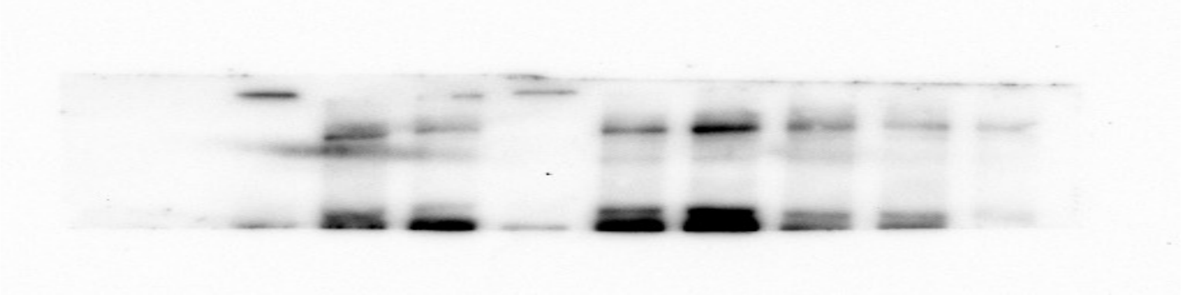

OR+TRFHFD	NR	NR+TRFSD	OR	OR+TRFHFD	OR+TRFSD	NR	OR	OR+TRFSD

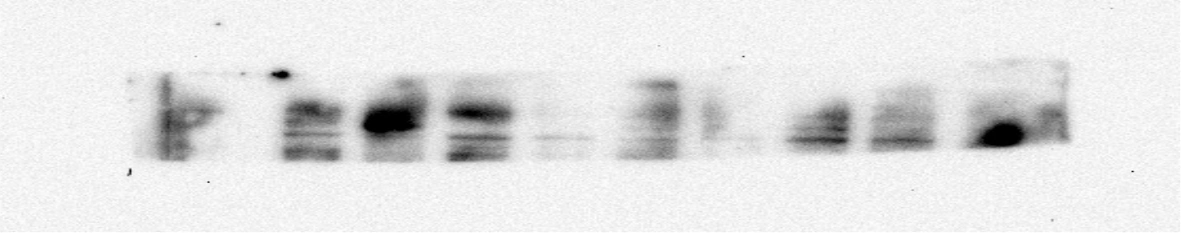

OR+TRFHFD	NR	NR+TRFSD	OR	OR+TRFHFD	OR+TRFSD	NR	OR	OR+TRFSD

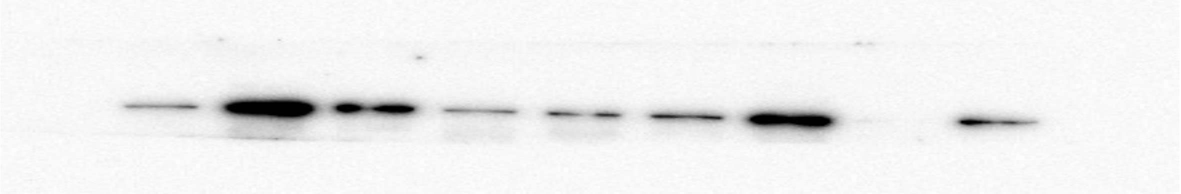

Akt

OR	NR	NR+TRFSD	OR	OR+TRFHFD	OR+TRFSD	NR	NR+TRFSD	OR+TRFSD

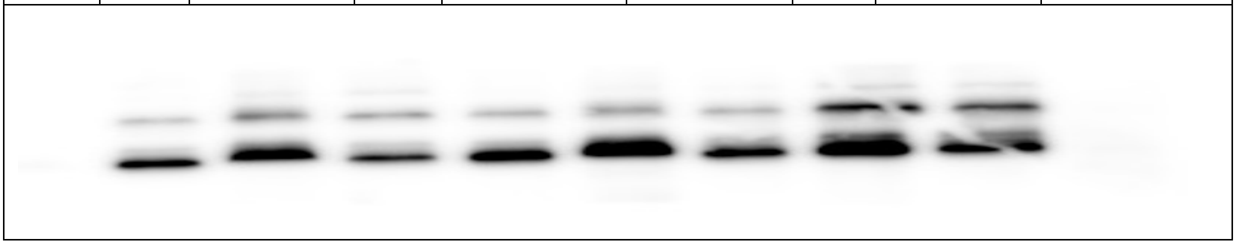

OR	OR+TRFHFD	OR+TRFSD	NR	NR	NR+TRFSD	OR	OR+TRFHFD	OR+TRFSD

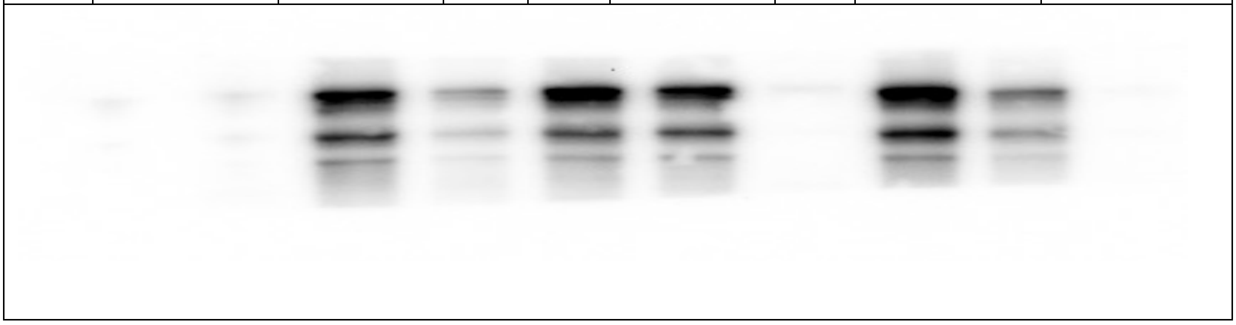

NR	NR+TRFSD	OR	OR+TRFHFD	OR+TRFSD	OR+TRFHFD	NR	NR+TRFSD	OR

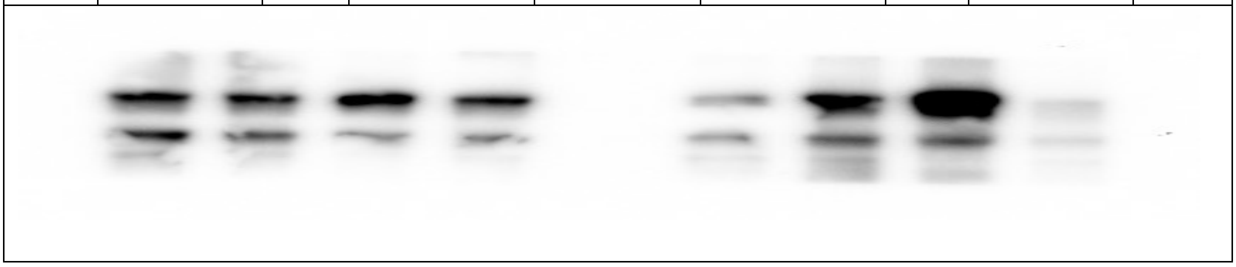

OR	OR+TRFHFD	OR+TRFSD	OR	NR	NR+TRFSD	OR	OR+TRFHFD	OR+TRFSD

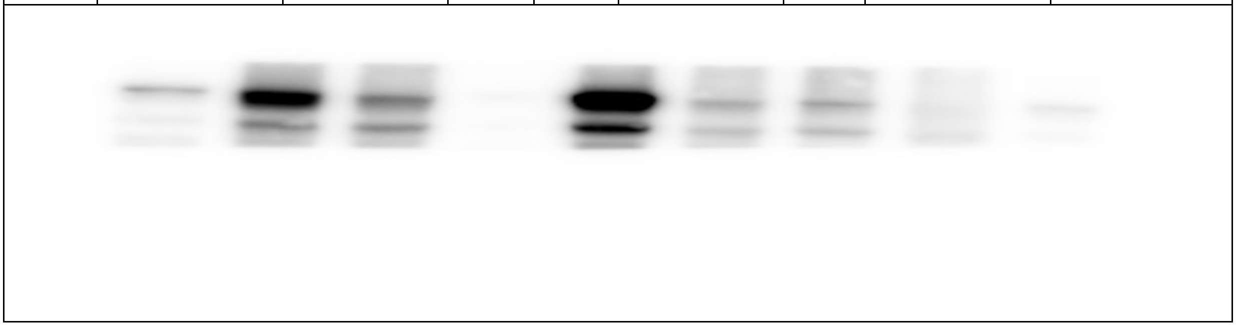

β-actin

NR	NR+TRFSD	OR	OR+TRFHFD	OR+TRFSD	OR	NR	NR+TRFSD	OR

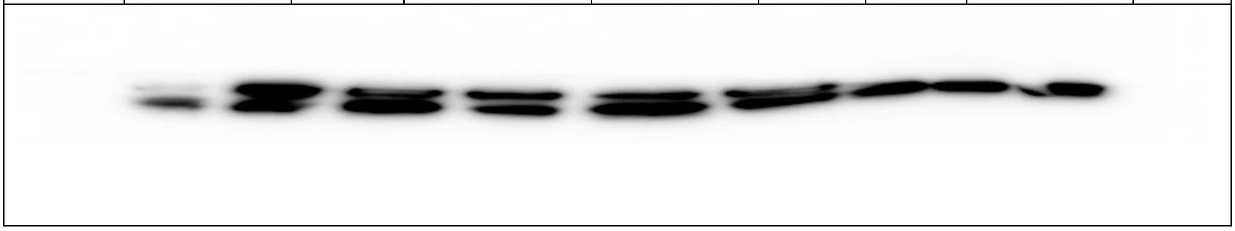

OR+TRFHFD	NR	NR+TRFSD	OR	OR+TRFHFD	OR+TRFSD	NR	OR	OR+TRFSD

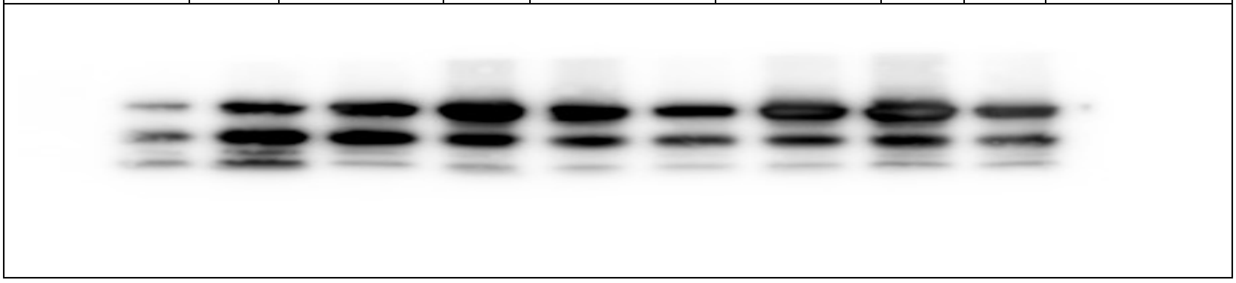

OR	OR+TRFHFD	OR+TRFSD	NR	NR	NR+TRFSD	OR	OR+TRFHFD	OR+TRFSD

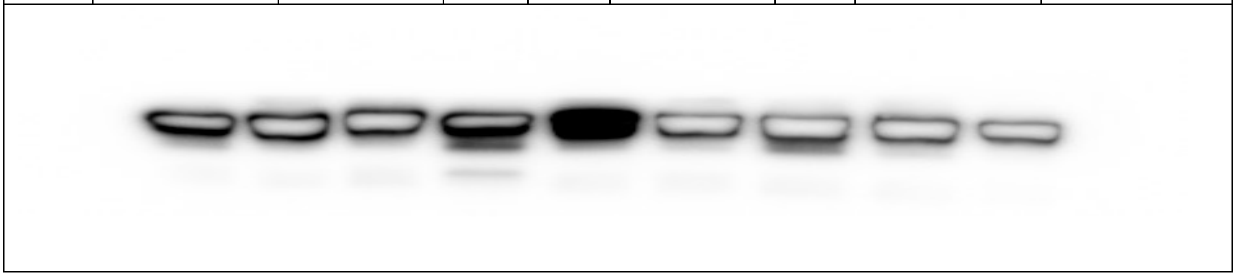

OR	NR	NR+TRFSD	OR	OR+TRFHFD	OR+TRFSD	NR	NR+TRFSD	OR

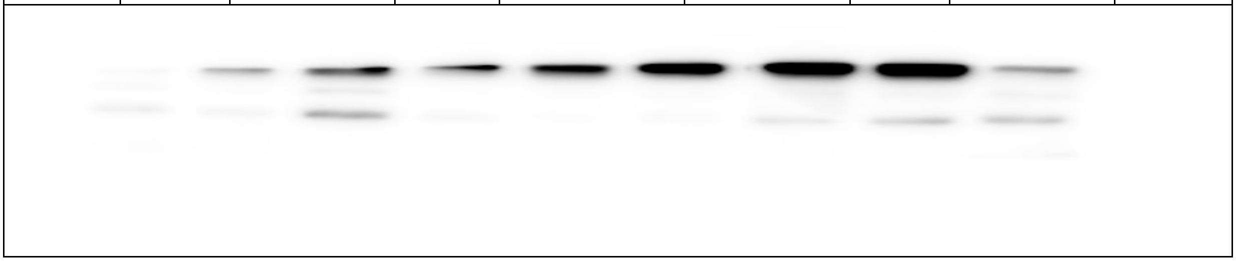

NR	NR+TRFSD	OR	OR+TRFHFD	OR+TRFSD	NR+TRFSD	NR	OR	OR+TRFSD

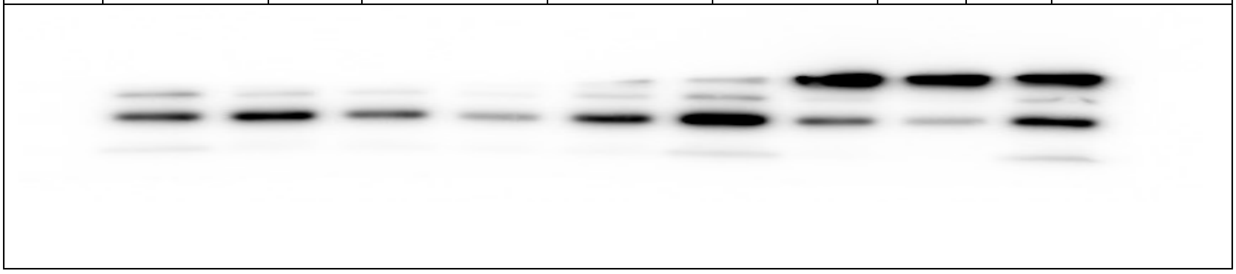

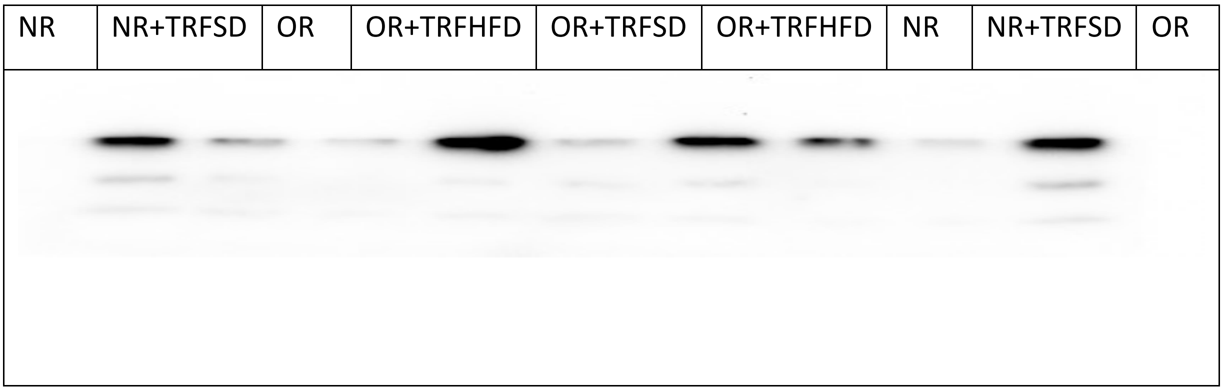

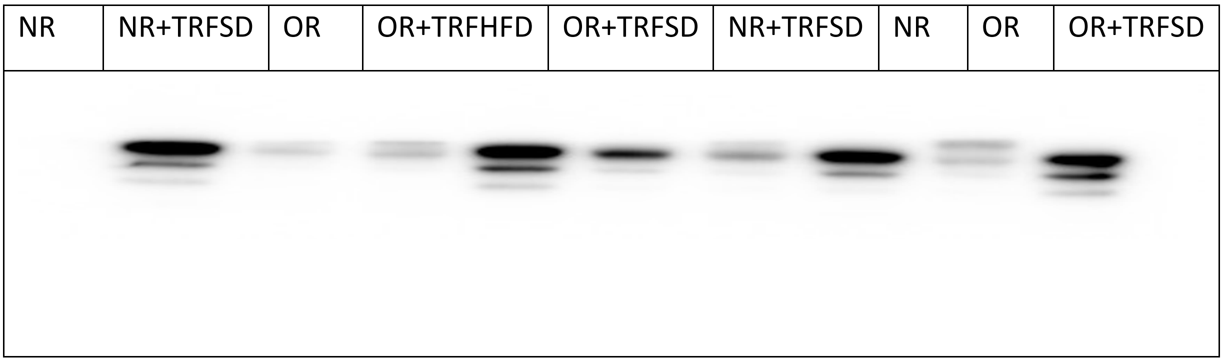

***Grouping**

Rats fed with standard commercial food pellets for 6 weeks + 6 weeks of standard commercial food pellets (NR)

Rats fed with standard commercial food pellets for 6 weeks + 6 weeks of TRF of standard commercial food pellets (NR+TRFSD)

Rats fed with HFD for 6 weeks + 6 weeks of HFD (OR)

Rats fed with HFD for 6 weeks + 6 weeks TRF of HFD (OR+TRFHFD)

Rats fed with HFD for 6 weeks + 6 weeks TRF of standard commercial food pellets (OR+TRFSD)
